# Diversity of the H9N2 Avian Influenza Virus in Shandong Province, China

**DOI:** 10.1155/tbed/1432483

**Published:** 2025-01-16

**Authors:** Ruixue Xue, Huiling Ma, Zixin Jiang, Linlin Xing, Guisheng Wang, Zouran Lan, Yue Zhang

**Affiliations:** Shandong Provincial Center for Animal Disease Control (Shandong Provincial Center for Zoonoses Epidemiology Investigation and Surveillance), 4566 Tangye West Road, Licheng District, Jinan 250100, China

**Keywords:** avian influenza virus, H9N2 subtype, phylogenetic analysis

## Abstract

H9N2 avian influenza virus (AIV) is one of the main pathogens causing respiratory disease in chicken; however, differentiating this virus from infectious bronchitis virus (IBV) and newcastle disease virus (NDV) only using clinical signs is difficult. In this study, 492 tracheal and lung tissue samples were collected from chicken farms in Shandong reporting respiratory symptoms and tested using Reverse Transcription-Polymerase Chain Reaction (RT-PCR) for the presence of H9N2 AIVs, IBVs, and NDVs. The H9N2 AIVs positive samples were inoculated with chicken embryos. Whole-genome sequences of the positive strains were obtained using Illumina MiSeq and analyzed for genetic evolution and key amino acid sites mutation. Seventy-two samples were positive for H9N2 subtype AIV, with a positive rate of 14.63%, while the positive rates of IBV and NDV were 6.10% and 0.41%, respectively. Thirty-four strains of H9N2 AIVs were obtained from positive samples. Phylogenetic tree analysis of HA and NA genes revealed that the 34 H9N2 AIV strains belonged to Y280-like and F/98-like branches, respectively. Clear temporalphylogenetic branching was observed, with some strains found in the “pre-2013 isolates” clade and others in the “post-2013 isolates” clade, which raised the possibility that strains in the former clade may have undergone recombination with viral strains from 10 years ago. Among the internal amino acid sites that are key to mammalian adaptation, all strains had an I368V mutation in the PB1 gene that enhanced viral transmissibility in mammals, and the PB2 genes of some strains were mutated to enhance the mammalian adaptation of I292V and A588V. Thus, the H9N2 AIV gene segments in Shandong have different degrees of recombination and gene variation, necessitating vigilant monitoring of virus variation.

## 1. Introduction

Respiratory disease of chicken, induced by bacteria or viruses, is a severe infectious syndrome that causes serious economic losses. H9N2 avian influenza virus (AIV) is an important etiologic agent of respiratory disease in the poultry-farming industry, but differentiating this virus from infectious bronchitis virus (IBV) and newcastle disease virus (NDV) only using clinical signs is difficult. The H9N2 AIV is often involved in mixed or secondary infections with other pathogenic microorganisms, leading to respiratory disorders, a slow growth rate, decreased egg production, and immune suppression in poultry [1]. Moreover, the H9N2 AIV has been detected in multiple avian species, including chickens, domestic waterfowl, and pigeons [2, 3]. Notably, H9N2 AIV in poultry populations has already acquired the ability to cross interspecies barriers and can directly infect mammals, even humans without a need for intermediate hosts, especially in China [4].

H9N2 AIV, a low pathogenic virus, has been circulating in China since 1993. Studies have reported that H9N2 reasortant-bearing genes from 2009 H1N1 pandemic virus exhibited increasing virulence in mice and transmissibility in ferrets [5]. Recent studies showed that most of H9N2 AIV circulating in China possesses the ability to bind *α*-2,6 mammalian sialic acid receptor. Moreover, most of the H9N2 isolates harbor an L at the 226 position of HA protein, which is the residue determining the receptor-binding property [6]. In addition, H9N2 AIV has donated internal genes to the human H5N1, H5N6, H7N9, and H10N8 subtypes [7–9], and hence could potentially trigger an influenza pandemic [10–12].

The Shandong province is a major poultry-farming region of China. However, the prevalence and molecular characterization of H9N2 AIV circulating in chickens in Shandong remain largely unknown. In order to understand the prevalence and genetic variation of H9N2 subtype avain influenza virus in Shandong, a total 492 tracheal and lung tissue samples were collected from chicken farms with respiratory symptoms in partial areas. Then H9N2 AIVs, IBVs, and NDVs were detected via RT-qPCR using amplication kits, and the H9N2 AIVs positive samples were inoculated with chicken embryos for two generations. Whole-genome sequences were sequenced of the H9N2 AIVs positive strains by applying Illumina Miaseq platform, and genetic evolution and mutation at positions associated with viral pathogenicity and transmissibility were analyzed to the variation of H9N2 AIV genes in Shandong province.

## 2. Materials and Methods

### 2.1. Sample Source and Viral Nucleic Acid Detection

Give priority to the poultry farms that have respiratory symptoms, a total of 492 chickens (6 to 10 chickens were collected from each farm) were collected from 60 poultry farms in different parts of Shandong (Jinan, Heze, Liaocheng, Linyi, Jining, Weifang, Dezhou, Binzhou, Taian, and Yantai) between January 2020 and December 2022.

All of the chickens were killed, as described previously [13]. Then, about 0.2 g of tracheal and lung tissues was collected using sterile scissors and tweezers in 1 mL of phosphate-buffered saline (PBS). Samples were centrifuged at 10,000×*g* for 5 min, and then 200-μL samples of the supernatant were used in the extraction using a MagNA Pure 96 Purification System with a MagNA Pure LC Total Nucleic Acid Isolation Kit (Roche, Basel, Switzerland). The isolated RNAs were stored at −80°C.

For diagnosis of H9N2 AIVs and IBVs, RT-qPCR was done using the amplification kit (Transgene, Beijing, China) with the specific primers [14, 15], and for NDV detection, the Reverse Transcription-Polymerase Chain Reaction (RT-PCR) method was performed using two pairs of primers [16].

### 2.2. Virus Isolation and Identification

Each H9N2 AIVs-positive tissue sample was placed in a transport medium that consisted of 1 mL of PBS with penicillin (2000 U/mL) and streptomycin (2000 μg/mL). Samples were centrifuged at 8000 rpm for 10 min, and the supernatant was inoculated into the allantoic cavity of 10-day-old specific pathogen-free embryonated chicken eggs and incubated for 72 h at 37°C. Chicken embryos that died within 24 h were discarded, and allantoic fluid was collected from the remaining embryos after 72 h of incubation. RNA was extracted from the allantoic fluid using MagNA Pure LC Total Nucleic Acid Isolation Kit, and H9N2 AIVs were detected via RT-qPCR. The H9N2 AIVs-positive allantoic fluid samples were stored at −80°C.

### 2.3. Viral-Genome Sequencing and Sequence Analysis

RNA extraction was performed on H9N2 AIVs-positive allantoic fluid samples, and the isolates were sent to Shanghai Tanpu Biotech Co., Ltd. for whole-genome sequencing on the Illumina MiSeq sequencing platform. MEGAX software was employed to perform phylogenetic analysis for each gene segment together with the reference strain, and DNASTAR MegAlign software was used to analyze key amino acid sites.

## 3. Results

### 3.1. RT-qPCR-Based Detection, H9N2 AIVs Isolation, and Virus Identification With Clinical Samples

**Figure 1:**
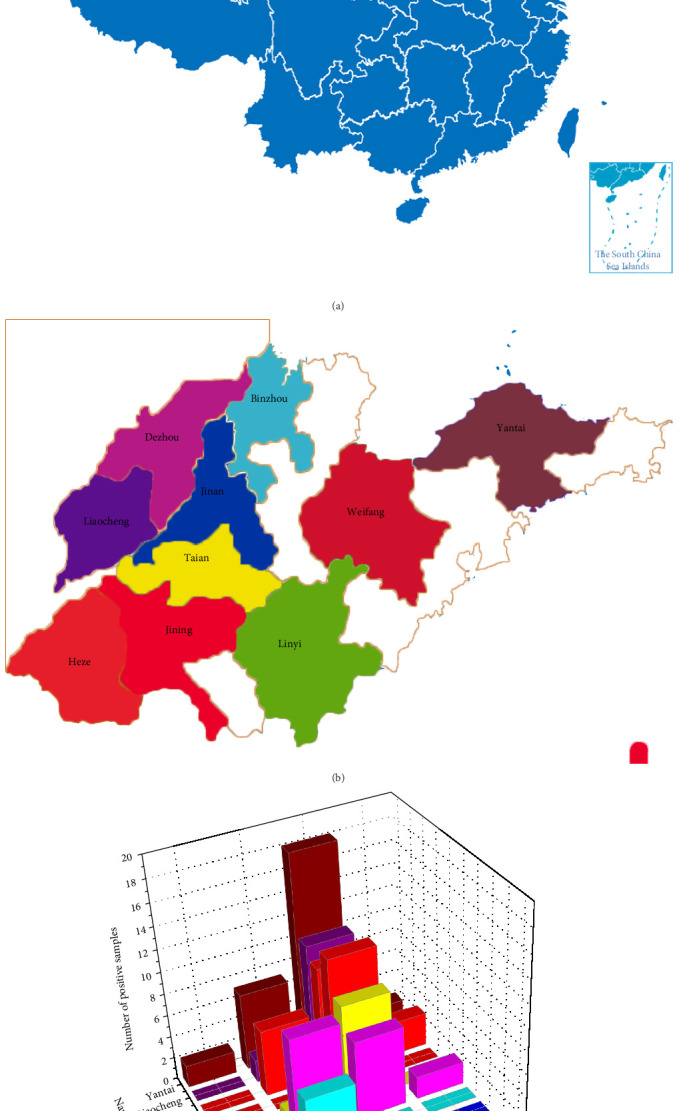
Number of H9N2-AIV, IBV, NDV positive samples collected from chicken farms in different cities of the Shandong province. (a) The map of China, with the Shandong region marked in red. (b) The map of Shandong, in which the cities containing chicken farms reporting respiratory symptoms are marked in different colors. (c) Number of H9N2-AIV, IBV, NDV, and H9N2-AIV/IBV coinfection positive samples in different cities in the Shandong province (The numbers above the bar chart represent the corresponding number of positive samples).

492 samples collected from 60 chicken farms in different parts of Shandong were tested for H9N2 AIVs, IBVs, and NDVs, which were performed using commercial kits. The results showed that 72 samples of 34 farms were positive for H9N2 AIV, with a positive rate of 14.63%; 30 samples of 10 farms were positive for IBV, with a positive rate of 6.10%; seven samples of four farms were positive for H9N2 AIV/IBV coinfection, with a positive rate of 1.42%; two samples of one farm were positive for NDV, with a positive rate of 0.41%. (Table 1 and Figure 1).

After inoculation in chicken embryos, 34 H9N2 AIVs strains selected from different farms were isolated (Table 2). All gene sequences were deposited in GenBank under the following accession numbers: OR528204–OR528237, OR528258–OR528291, OR528297–OR528330, OR528340–OR528373, OR528409–OR5284442, OR528456–OR528489, OR528497–OR528530, and OR528554–OR528587.

### 3.2. Analysis of the H9N2 Viral Genome and Key Amino Acid Sites

#### 3.2.1. Analysis of the HA gene Sequence and Key Amino Acid Sites

**Figure 2:**
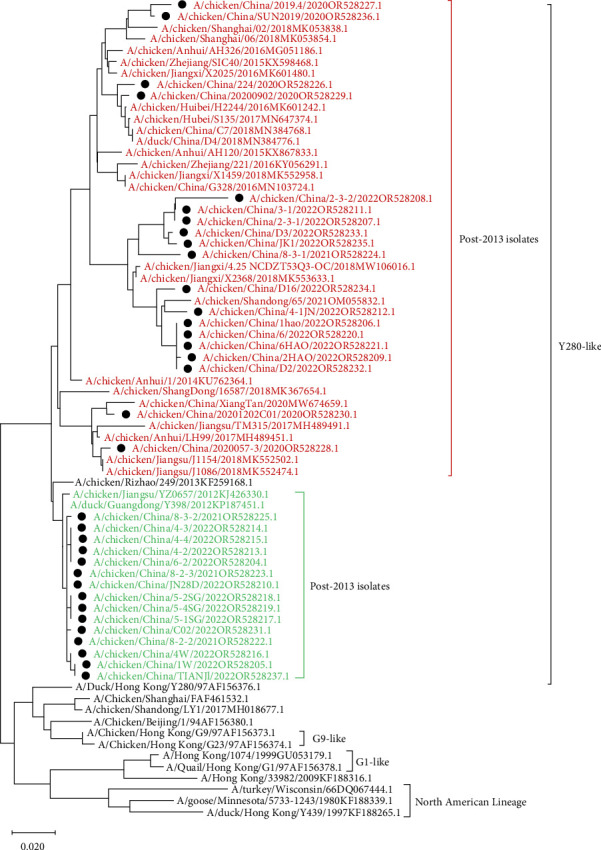
Phylogenetic tree based on the HA gene (The length of the gene fragment was 1683 bp, ATG nt~TAA nt). The tree was generated by neighbor-joining method using MEGAX software. Phylogenetic trees were based on the comparison of nucleotide sequences of the H9N2 AIVs isolated in this study to the reference AIV sequences published in GenBank. All of those isolates were presented in black circles, in which post-2013 isolates and pre-2013 isolates were highlighted in red and green, respectively. The scale bar represents the distance unit between sequence pairs.

Compared with the reference strains, HA genes of all 34 isolates belonged to the Y280-like clade, with two smaller, independent clades in time, 17 isolates in the “pre-2013 isolates” clade, and 17 isolates in the “post-2013 isolates” clade (Figure 2).

In all 34 H9N2 isolates, the HA cleavage site was consistent with the characteristics of LPAIV (low pathogenic avian influenza), with a discontinuous basic amino acid sequence of 333PSRSSR↓GLF341. The Q226L amino acid site mutation confers the ability of AIV to bind with *α*-2, 6 receptors. The HA receptor-binding domain of H9N2 AIV forms a ribbon-like structure at the top of the protein, and amino acid changes in this region can alter the viral receptor-binding properties. In the above region, the 109, 161, 202, 203, 234, and 236 amino acid sites of the 34 isolates were relatively conserved, whereas the 163T/N and 191N/H amino acid sites have two alternatives, and the 198V/T/A amino acid site has three alternatives (Table 3).

The N-glycosylation site of HA in H9N2 AIVs significantly impacts its pathogenicity. Among the 34 isolates, the HA amino acid sequences of 33 isolates contained seven potential glycosylation sites, and one isolate contained six glycosylation sites. The glycosylation sites of all isolates were relatively conserved at 29, 141, and 492 amino acid sites, compared with those found in the reference strain. The T220I/V amino acid site mutation caused all isolates to lack a potential glycosylation site at amino acid 218. In addition, the P315S amino acid site mutation provided a potential glycosylation site at amino acid 313 in all isolates (Table 4).

#### 3.2.2. Analysis of the NA Gene Sequence and Key Amino Acid Sites

**Figure 3:**
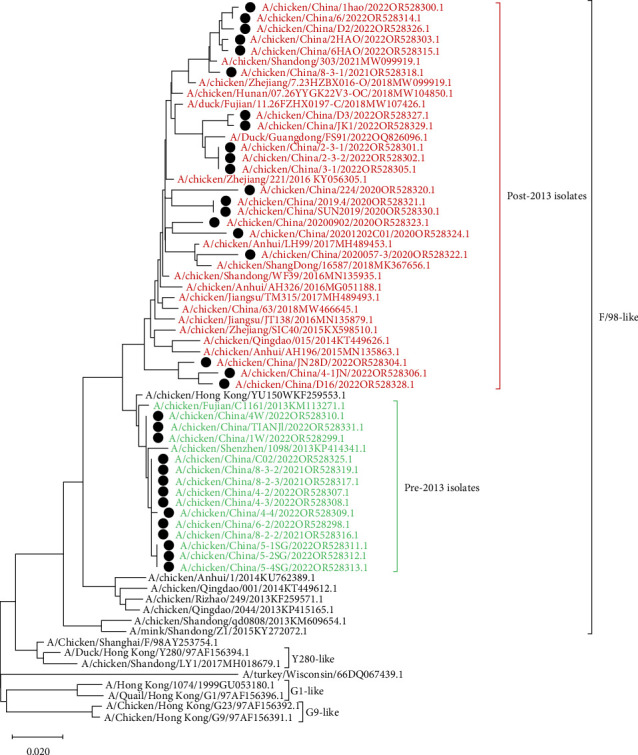
Phylogenetic tree based on the NA gene (The length of the gene fragment was 1398 bp, ATG nt~ATA nt). The tree was generated by neighbor-joining method using MEGAX software. Phylogenetic trees were based on the comparison of nucleotide sequences of the H9N2 AIVs isolated in this study to the reference AIV sequences published in GenBank. All of those isolates were presented in black circles, in which post-2013 isolates and pre-2013 isolates were highlighted in red and green, respectively. The scale bar represents the distance unit between sequence pairs.

Compared with the reference strains, NA genes of all 34 isolates belonged to the F/98-like clade, with two smaller, independent clades in time, 16 isolates in the “pre-2013 isolates” clade, and 18 isolates in the “post-2013 isolates” clade (the NA and HA gene of the A/chicken/China/JN28D/2022 virus belonged to the “post-2013 isolate” clade and the “pre-2013 isolate” clade, respectively; Figure 3).

Erythrocyte sites on the NA surfaces of influenza viruses are composed of 366–373, 399–404, and 431–433 amino acid sites, which play critical roles in the replication and invasion of influenza viruses. Our analysis revealed that the 366–373 and 399–404 amino acid sites were relatively variable in the 34 isolates, with 369, 401, 402, and 403 amino acid sites exhibiting the highest numbers of mutations. In contrast, 431–433 amino acid sites were relatively conserved (Table 5).

Among the 34 isolates, the N-glycosylation site of NA occurred most frequently at the 69, 86, 146, 200, 234, and 368 amino acid sites. The A/chicken/China/4-1JN/2022 isolate contained an S70N mutation, which resulted in the deletion of one glycosylation site. In summary, 29 strains contained six potential glycosylation sites, and five strains contained seven potential glycosylation sites.

#### 3.2.3. Analysis of Internal Sequences and Key Amino Acid Sites

**Figure 4:**
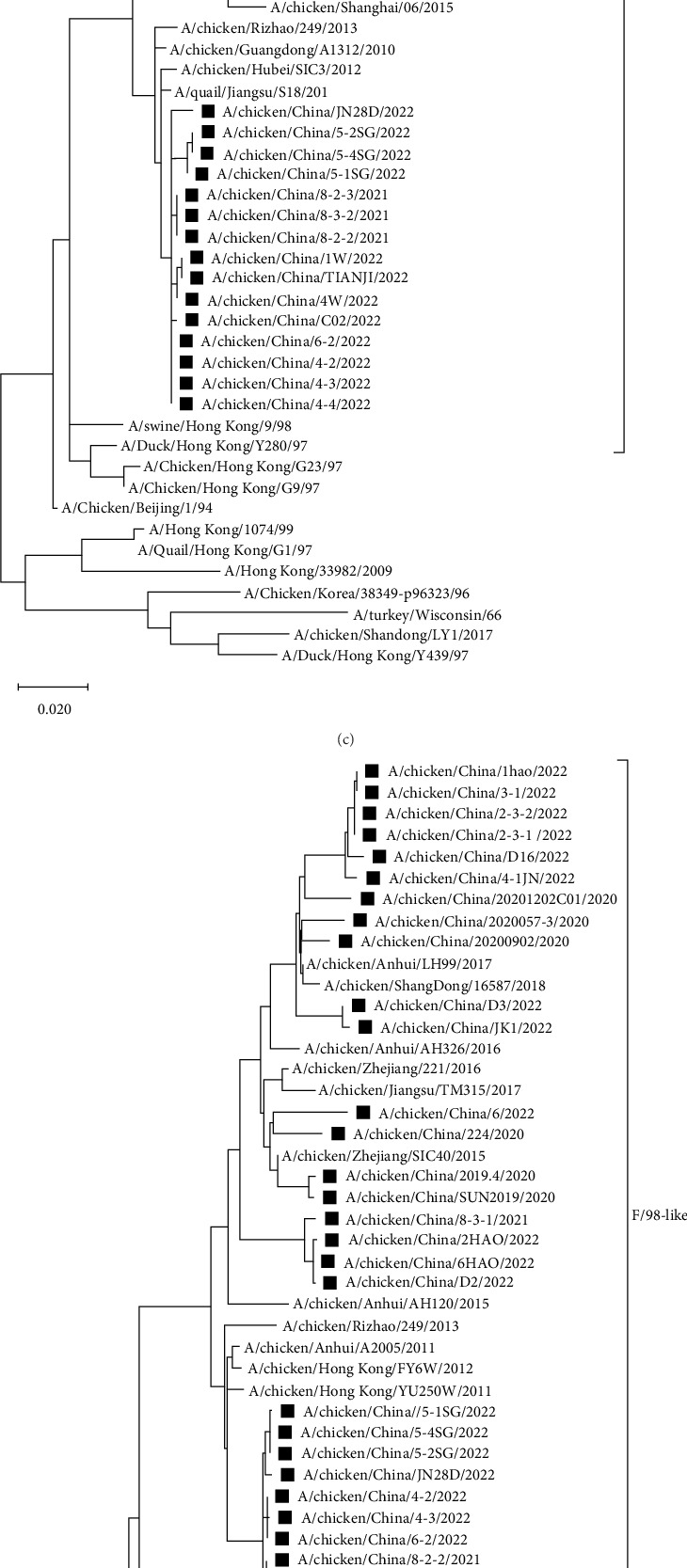
Phylogenetic trees based on the PB1, PB2, PA, NP, M, and NS genes (The complete ORF of each gene fragment was used for evolutionary analysis). The tree was generated by neighbor-joining method using MEGAX software. Phylogenetic trees were based on the comparison of nucleotide sequences of the H9N2 AIVs isolated in this study to the reference AIV sequences published in GenBank. Isolates are highlighted in black squares. The scale bar represents the distance unit between sequence pairs.

Phylogenetic analysis of internal genes against the reference strains revealed that the PB1, PA, and NP genes of the 34 isolates belonged to the F/98-like clade, that the PB2 and M genes belonged to the G1-like clade, and that the NS gene belonged to the Y280-like clade (Figure 4). Thus, the internal genes of H9N2 AIVs in Shandong have undergone recombination to varying extents, resulting in the production of different types of H9N2 AIVs.

Previous studies showed that amino acid site changes in the PB2 protein, e.g., I292V, A588V, E627K, and D701N can increase viral infectivity and pathogenicity in mammals [17–20]. Our analysis of key amino acid sites in the PB2 proteins of all 34 H9N2 isolates revealed that the PB2 proteins of 33 strains carried the I292V mutation, that those of 19 strains carried the A588V mutation, and that those of two strains carried the E627V mutation. All isolates were relatively conserved at amino acid site 701. The I368V mutation in PB1 can enhance AIV transmission in ferrets [21], and all 34 isolates in this study exhibited the I368V mutation in PB1.

## 4. Discussion

Although H9N2 AIV is one of the main pathogens causing respiratory disease in chickens, differentiating it from IBV and NDV only based on clinical signs is difficult. In this study, 14.63% of the investigated chicken farms were positive for H9N2 AIV, and 6.10% and 0.41% were positive for IBV and NDV, respectively, which indicated that H9N2 AIV is a major pathogen causing chicken respiratory disease in Shandong province.

As the predominant LPAIV in China, H9N2 AIV not only endangers the robust development of the poultry farming industry but also poses potential threats in terms of human transmission [22, 23]. By analyzing the evolutionary tree of the HA and NA genes of the 34 H9N2 AIV strains, we found that the HA and NA genes belonged to the Y280-like branch and the F/98-like branch, respectively. Furthermore, phylogenetic analysis of the HA and NA genes revealed significant temporal phylogenetic branching in the 34 isolates, with some strains found in the “pre-2013 isolates” clade and others in the “post-2013 isolates” clade. These data raise the possibility that strains in the former clade may have undergone recombination with viral strains from 10 years ago, which implies that the H9N2 AIV strains from 10 years ago are still circulating in the environment. Hence, we should analyze the neutralization capacity of current vaccines to these strains. Subsequently, if the neutralizing ability cannot achieve sufficient protective effect, those vaccines should be replaced with a new vaccine that could prevent both “pre-2013 isolates” and “post-2013 isolates” viruses. Meanwhile, all of the 34 H9N2 AIV strains corresponded to chickens that were immunized with the H9 subtype AIV vaccines, which indicated that H9N2 AIVs were still prevalent in immunized flocks, with the possible reason of antigenic difference between pandemic strains and vaccine strains of H9N2 AIVs leading to current vaccines lacking adequate protection against H9N2 AIVs. Therefore, the development of a universal vaccine with broad-spectrum neutralizing activity is of great significance for the control of H9N2 AIVs. Phylogenetic analysis of the internal genes revealed that the PB1, PA, and NP genes of all 34 isolates belonged to the F/98-like clade; the PB2 and M genes belonged to the G1-like clade; and the NS gene belonged to the Y280-like clade. Thus, the internal genes of H9N2 AIV in Shandong have undergone recombination to varying extents, resulting in different types of H9N2 AIVs, which can act as gene donors for other AIV subtypes to produce recombinant viruses such as H7N9, H5N6, H5N1, in special, H3N8 and H10N3 AIVs [24, 25], which not only exist widely among chicken flocks but have also been detected in the human population in recent years [26], posing potential threats to human health [27].

HA is the main surface protein and protective antigen of AIV, which determines pathogenicity and host specificity. Furthermore, mutations at key amino acid sites in this protein can directly affect the antigenicity, pathogenicity, host specificity, and other properties of the virus [28]. The HA amino acid Q226L mutation in all 34 strains of AIV isolated in this study endowed these viruses with the ability to bind with mammalian *α*-2,6 sialic acid receptors. Previous findings demonstrated that the HA sialic acid mutations of I163T, H191N, A198T/V, Q226L, and G236S in H9N2 AIV can increase the receptor-binding ability of AIV [29]. The isolates in this study showed HA mutations at the 163, 191, and 918 sialic acid sites, which can enhance the receptor-binding ability of the virus. Both the addition or deletion of N-glycosylation sites in the HA protein can lead to changes in AIV antigenicity and pathogenicity [30, 31]. Analysis of the HA N-glycosylation sites in the 34 isolates revealed that the T220I/V mutation led to the deletion of one glycosylation site at the 218 sialic acid site, whereas the HA P315S mutation led to the addition of a potential glycosylation site at the 313 sialic acid site in some isolates. The deletion or addition of these sites may contribute to the antigenicity and pathogenicity of the isolates.

NA is another important glycoprotein in AIV, and glycosylation sites in NA play crucial roles in NA protein composition and viral replication. The molecular characteristics analysis of the NA protein in the 34 isolates revealed that the glycosylation sites of most strains were located at the 69, 86, 146, 200, 234, and 368 amino acid sites. The A/chicken/China/4-1JN/2022 strain acquired an S70N mutation, which led to the deletion of one glycosylation site; however, further investigation is needed to determine whether this mutation can impact the biological function of AIV.

In addition to the key amino acid sites mentioned above, the internal genes of H9N2 AIV also contain several key amino acid sites that can affect host adaptability. For example, I292V, A588V, E627K, and D701N amino acid site mutations in the PB2 protein can increase viral infectivity and pathogenicity in mammals [17–19, 32]. Among the 34 isolates found in this study, 33 carried the I292V mutation in PB2, and 19 carried the A588V mutation, indicating that these viral strains may have enhanced adaptability with respect to mammals. Furthermore, all isolates exhibited the I368V mutation in PB1, which further demonstrates that H9N2 AIV is continually adapting to replicate in mammals.

Overall, our results demonstrated that H9N2 AIV is circulating in Shandong, China. Clear temporal phylogenetic branching and different phylogenetic types of H9N2 AIV revealed the variability and recombination complexity of H9N2 AIV in the Shandong province. Furthermore, mutations in numerous amino acid sites occurred in H9N2 AIV, particularly the mammalian-adaptive amino acid sites, which indicated that further investigation is needed to verify the impact of these amino acid changes on viral transmissibility and pathogenicity in mammals. Overall, our study underscores the importance of continued surveillance and monitoring of H9N2 AIVs, particularly in regions where these viruses are prevalent, to identify and address emerging threats to both animal and human health.

## Figures and Tables

**Table 1:** Positive samples information in different cities.

City	No. of samples/farms	No. of H9N2 AIV positive samples/farms	No. of IBV positive samples/farms	No. of H9N2 AIV/IBV samples/farms	No. of NDV positive samples/farms
Yantai	138/16	18/9	7/2	2/1	2/1
Liaocheng	62/8	12/6	2/1	0	0
Jining	102/11	11/5	6/2	3/2	0
Weifang	62/9	13/7	0	0	0
Taian	41/4	7/2	1/1	0	0
Dezhou	39/4	6/2	10/3	2/1	0
Binzhou	20/2	0	4/1	0	0
Jinan	10/1	1/1	0	0	0
Linyi	9/1	2/1	0	0	0
Heze	9/1	2/1	0	0	0

**Table 2:** Specific information of H9N2 AIVs isolation.

H9N2 AIVs	Collection time	Area	H9N2 AIVs	Collection time	Area
A/chicken/China/2HAO/2022	2022.04	Yantai	A/chicken/China/2020057-3/2020	2020.05	Liaocheng
A/chicken/China/6HAO/2022	2022.01	Yantai	A/chicken/China/20201202C01/2020	2020.12	Liaocheng
A/chicken/China/D2/2022	2022.05	Jining	A/chicken/China/8-2-3/2021	2021.12	Yantai
A/chicken/China/1hao/2022	2022.04	Yantai	A/chicken/China/C02/2022	2022.05	Yantai
A/chicken/China/6/2022	2022.04	Jining	A/chicken/China/8-2-2/2021	2021.12	Jining
A/chicken/China/D16/2022	2022.05	Liaocheng	A/chicken/China/8-3-2/2021	2021.10	Liaocheng
A/chicken/China/4-1JN/2022	2022.01	Liaocheng	A/chicken/China/6-2/2022	2022.01	Dezhou
A/chicken/China/8-3-1/2021	2021.12	Dezhou	A/chicken/China/4-2/2022	2022.01	Jinan
A/chicken/China/D3/2022	2022.05	Heze	A/chicken/China/4-3/2022	2022.01	Yantai
A/chicken/China/JK1/2022	2022.03	Weifang	A/chicken/China/4-4/2022	2022.01	Yantai
A/chicken/China/3-1/2022	2022.02	Weifang	A/chicken/China/1W/2022	2022.04	Liaocheng
A/chicken/China/2-3-1/2022	2022.06	Taian	A/chicken/China/TIANJI/2022	2022.04	Weifang
A/chicken/China/2-3-2/2022	2022.06	Taian	A/chicken/China/4W/2022	2022.04	Jining
A/chicken/China/20200902/2020	2020.09	Weifang	A/chicken/China/JN28D/2022	2022.02	Jining
A/chicken/China/224/2020	2020.01	Yantai	A/chicken/China/5-1SG/2022	2022.07	Weifang
A/chicken/China/2019.4/2020	2020.01	Yantai	A/chicken/China/5-2SG/2022	2022.07	Weifang
A/chicken/China/SUN2019/2020	2020.01	Yantai	A/chicken/China/5-4SG/2022	2022.07	Weifang

**Table 3:** Analysis of key amino acid sites of HA gene (H9 numbering^a^).

Viruses	Receptor-binding sites								
	109	161	163	191	198	202	203	234	236
CK/BJ/1/94	Y	W	T	N	V	L	Y	Q	G
DK/HK/Y280/97	Y	W	T	N	T	L	Y	L	G
DK/HK/Y439/97	Y	W	T	H	E	L	Y	Q	G
QU/HK/G1/97	Y	W	T	H	E	L	Y	L	G
CK/SH/F/98	Y	W	T	N	A	L	Y	Q	G
Isolates (ratio)	Y	W	T (21/34) N (13/34)	N (33/34) H (1/34)	V (27/34) T (5/34) A (2/34)	L	Y	L	G

^a^The whole length of amino acid sequence of HA prorein.

**Table 4:** Analysis of potential glycosylation sites of HA gene (H9 numbering).

Viruses	Potential glycosylation sites									
	29 NST	141 NVS	145 NGT	166 NNT	218 NRT	298 NTT	305 NVS	313 NCS	492 NGT	551 NGS
CK/BJ/1/94	√	NVT	×	×	√	√	√	×	√	√
DK/HK/Y280/97	√	√	×	×	√	√	√	×	√	√
DK/HK/Y439/97	√	√	×	×	√	√	√	×	√	√
QU/HK/G1/97	√	NVT	×	×	√	NST	NIT	×	√	√
CK/SH/F/98	√	√	×	×	√	√	√	×	√	√
Isolates (ratio)	√	√	×	×	×	√	√ (28) NIT (6)	√	√	× (1) √ (33)

*Note:* √ indicates the existence of a potential N-glycosylation site; × indicates the absence of a potential N-glycosylation site.

**Table 5:** Distribution of erythrocyte-binding sites of NA gene.

Proportion of virus	Erythrocyte-binding sites		
	366–373	399–404	431–433
19/34	IKNGSRSG	DSDDWS	PQE
4/34	IKNGSRSG	DSDDLS	PHE
1/34	IKNSSRSG	DSENWS	PQE
1/34	IKNSSRSG	DGDDLS	PHE
1/34	IKNSSRSG	DSVDWS	PQE
2/34	IKNGSRSG	DSVDWS	PQE
1/34	IKNGSRSG	DSDDWT	PQE
3/34	IKNSSRSG	DSDNWS	PQE
2/34	IKNGSRSG	DSDDLS	PQE

## Data Availability

The data underlying this article are available in GenBank under the following accession numbers: OR528204–OR528237, OR528258–OR528291, OR528297–OR528330, OR528340–OR528373, OR528409–OR5284442, OR528456–OR528489, OR528497–OR528530, and OR528554–OR528587. The data are freely accessible and can be downloaded for further analysis and verification.

## References

[1] Butt K. M., Smith G. J. D., Chen H. (2005). Human Infection With an Avian H9N2 Influenza A Virus in Hong Kong in 2003. *Journal of Clinical Microbiology*.

[2] Sun Y., Liu J. (2015). H9N2 Influenza Virus in China: A cause of Concern. *Protein & Cell*.

[3] Jiang W., Liu S., Hou G. (2012). Chinese and Global Distribution of H9 Subtype Avian Influenza Viruses. *PLoS One*.

[4] Taubenberger J. K., Kash J. C. (2010). Influenza Virus Evolution, Host Adaptation, and Pandemic Formation. *Cell Host & Microbe*.

[5] Jiang W., Liu S., Hou G. (2011). High Genetic Compatibility and Increased Pathogenicity of Reassortants Derived From Avian H9N2 and Pandemic H1N1/2009 Influenza Viruses. *Proceedings of the National Academy of Sciences of the United States of America*.

[6] Jiang W., Liu S., Hou G. (2016). A Single Mutation at Position 190 in Hemagglutinin Enhances Binding Affinity for Human Type Sialic Acid Receptor and Replication of H9N2 Avian Influenza Virus in Mice. *Journal of Virology*.

[7] Chen H., Yuan H., Gao R. (2014). Clinical and Epidemiological Characteristics of a Fatal Case of Avian Influenza A H10N8 Virus Infection: A Descriptive Study. *The Lancet*.

[8] Yu X., Jin T., Cui Y. (2014). Influenza H7N9 and H9N2 Viruses: Coexistence in Poultry Linked to Human H7N9 Infection and Genome Characteristics. *Journal of Virology*.

[9] Kandeil A., El-Shesheny R., Maatouq A. M. (2014). Genetic and Antigenic Evolution of H9N2 Avian Influenza Viruses Circulating in Egypt Between 2011 and 2013. *Archives of Virology*.

[10] Lin Y. P., Shaw M., Gregory V. (2000). Avian-to-Human Transmission of H9N2 Subtype Influenza A Viruses: Relationship Between H9N2 and H5N1 Human Isolates. *Proceedings of the National Academy of Sciences of the United States of America*.

[11] Lv J., Wei B., Yang Y. (2012). Experimental Transmission in Guinea Pigs of H9N2 Avian Influenza Viruses From Indoor Air of Chicken Houses. *Virus Research*.

[12] Zhang K., Zhang Z., Yu Z. (2013). Domestic Cats and Dogs are Susceptible to H9N2 Avian Influenza Virus. *Virus Research*.

[13] Ho P. S., Kwang J., Lee Y. K. (2005). Intragastric Administration of *Lactobacillus casei* Expressing Transmissible Gastroentritis Coronavirus Spike Glycoprotein Induced Specific Antibody Production. *Vaccine*.

[14] Monne I., Ormelli S., Salviato A. (2008). Development and Validation of a One-Step Real-Time PCR Assay for Simultaneous Detection of Subtype H5, H7, and H9 Avian Influenza Viruses. *Journal of Clinical Microbiology*.

[15] Callison S. A., Hilt D. A., Boynton T. O. (2006). Development and Evaluation of a Real-Time Taqman RT-PCR Assay for the Detection of Infectious Bronchitis Virus From Infected Chickens. *Journal of Virological Methods*.

[16] Kant A., Koch G., Roozelaar D. J. V., Balk F., Huurne A. T. (1997). Differentiation of Virulent and Non-Virulent Strains of Newcastle Disease Virus Within 24 Hours by Polymerase Chain Reaction. *Avian Pathology*.

[17] Sang X., Wang A., Chai T. (2015). Rapid Emergence of a PB2-E627K Substitution Confers a Virulent Phenotype to an H9N2 Avian Influenza Virus During Adoption in Mice. *Archives of Virology*.

[18] Gao W., Zu Z., Liu J. (2019). Prevailing I292V PB2 Mutation in Avian Influenza H9N2 Virus Increases Viral Polymerase Function and Attenuates IFN-*β* Induction in Human Cells. *Journal of General Virology*.

[19] Xiao C., Ma W., Sun N. (2016). PB2-588V Promotes the Mammalian Adaptation of H10N8, H7N9 and H9N2 Avian Influenza Viruses. *Scientific Reports*.

[20] Li Q., Wang X., Sun Z. (2015). Adaptive Mutations in PB2 Gene Contribute to the High Virulence of a Natural Reassortant H5N2 Avian Influenza Virus in Mice. *Virus Research*.

[21] Herfst S., Schrauwen E. J. A., Linster M. (2012). Airborne Transmission of Influenza A/H5N1 Virus Between Ferrets. *Science*.

[22] Bi Y., Li J., Li S. (2020). Dominant Subtype Switch in Avian Influenza Viruses During 2016–2019 in China. *Nature Communications*.

[23] Guo J., Wang Y., Zhao C. (2021). Molecular Characterization, Receptor Binding Property, and Replication in Chickens and Mice of H9N2 Avian Influenza Viruses Isolated From Chickens, Peafowls, and Wild Birds in Eastern China. *Emerging Microbes & Infections*.

[24] Islam1 A., Islam S., Flora M. S. (2023). Epidemiology and Molecular Characterization of Avian Infuenza A Viruses H5N1 and H3N8 Subtypes in Poultry Farms and Live Bird Markets in Bangladesh. *Scientific Reports*.

[25] Zhang Y., Shi J., Cui P. (2023). Genetic Analysis and Biological Characterization of H10N3 Influenza A Viruses Isolated in China From 2014 to 2021. *Journal of Medical Virology*.

[26] Sun H., Li H., Tong Q. (2023). Airborne Transmission of Human-Isolated Avian H3N8 Influenza Virus Between Ferrets. *Cell*.

[27] Islam A., Islam S., Amin E. (2022). Assessment of Poultry Rearing Practices and Risk Factors of H5N1 and H9N2 Virus Circulating Among Backyard Chickens and Ducks in Rural Communities. *PLoS One*.

[28] Jin X., Zha Y., Hu J. (2020). New Molecular Evolutionary Characteristics of H9N2 Avian Influenza Virus in Guangdong Province, China. *Infection, Genetics and Evolution*.

[29] Sun X., Belser J. A., Maines T. R. (2020). Adaptation of H9N2 Influenza Viruses to Mammalian Hosts: A Review of Molecular Markers. *Viruses*.

[30] Tsuchiya E., Sugawara K., Hongo S., Matsuzaki Y., Muraki Y., Nakamura K. (2002). Role of Overlapping Glycosylation Sequons in Antigenic Properties, Intracellular Transport and Biological Activities of Influenza A/H_2_N_2_ Virus Haemagglutinin. *Journal of General Virology*.

[31] Schulze I. T. (1997). Effects of Glycosylation on the Properties and Functions of Influenza Virus Hemagglutinin. *The Journal of Infectious Diseases*.

[32] Li X., Cui P., Zeng X. (2019). Characterization of Avian Influenza H_5_N_3_ Reassortants Isolated From Migratory Waterfowl and Domestic Ducks in China From 2015 to 2018. *Transboundary and Emerging Diseases*.

